# Impacts of salinity stress on crop plants: improving salt tolerance through genetic and molecular dissection

**DOI:** 10.3389/fpls.2023.1241736

**Published:** 2023-09-15

**Authors:** Kousik Atta, Saptarshi Mondal, Shouvik Gorai, Aditya Pratap Singh, Amrita Kumari, Tuhina Ghosh, Arkaprava Roy, Suryakant Hembram, Dinkar Jagannath Gaikwad, Subhasis Mondal, Sudip Bhattacharya, Uday Chand Jha, David Jespersen

**Affiliations:** ^1^ ICAR-Indian Agricultural Research Institute, New Delhi, India; ^2^ Bidhan Chandra Krishi Viswavidyalaya, Mohanpur, West Bengal, India; ^3^ Department of Crop and Soil Sciences, University of Georgia, Griffin, GA, United States; ^4^ School of Agriculture, GIET University, Gunupur, Rayagada, Odisha, India; ^5^ ICAR- National Institute of Biotic Stress Management, Raipur, India; ^6^ WBAS (Research), Government of West Bengal, Field Crop Research Station, Burdwan, India; ^7^ Centurion University of Technology and Management, Paralakhemundi, Odisha, India; ^8^ Indian Institute of Pulses Research, Kanpur, India

**Keywords:** salinity stress, oxidative stress, antioxidants, phenomics, QTL mapping, GWAS, genomic selection

## Abstract

Improper use of water resources in irrigation that contain a significant amount of salts, faulty agronomic practices such as improper fertilization, climate change etc. are gradually increasing soil salinity of arable lands across the globe. It is one of the major abiotic factors that inhibits overall plant growth through ionic imbalance, osmotic stress, oxidative stress, and reduced nutrient uptake. Plants have evolved with several adaptation strategies at morphological and molecular levels to withstand salinity stress. Among various approaches, harnessing the crop genetic variability across different genepools and developing salinity tolerant crop plants offer the most sustainable way of salt stress mitigation. Some important major genetic determinants controlling salinity tolerance have been uncovered using classical genetic approaches. However, its complex inheritance pattern makes breeding for salinity tolerance challenging. Subsequently, advances in sequence based breeding approaches and functional genomics have greatly assisted in underpinning novel genetic variants controlling salinity tolerance in plants at the whole genome level. This current review aims to shed light on physiological, biochemical, and molecular responses under salt stress, defense mechanisms of plants, underlying genetics of salt tolerance through bi-parental QTL mapping and Genome Wide Association Studies, and implication of Genomic Selection to breed salt tolerant lines.

## 1 Introduction

An abiotic or biotic constraint that reduces a plant’s ability to convert energy to biomass can be called plant stress (Grime, 1977). Reduction in crop yield due to various abiotic stresses such as excessive salt, drought, cold, and heat is a major challenge to meet rising food demand (Vorasoot et al., 2003; Kaur et al., 2008; Shanker and Venkateswarlu, 2011; Ahmad et al., 2012; Mantri et al., 2012; He et al., 2018). Crop cultivation is facing many challenges due to soil salinity which is not only limited to coastal areas, but also induced by other factors like faulty agronomic practices or the use of recycled water in irrigation that may contain large amounts of salts (Kumar and Sharma, 2020). Salinity stress and thereby the ensued damages to the plant may arise due to an excess accumulation of soluble ions (Na^+^, Ca^+^, K^+^, Mg^2+^) in the root zone (Tester and Davenport, 2003; Munns, 2005; Atta et al., 2022a; Atta et al., 2022b). Saline soils inevitably have salt concentrations high enough in their solutions to impair plant growth. Sulphates and chlorides of calcium, sodium, and magnesium are commonly associated with the development of soil salinity. However, the detrimental effects of Na salts on plants were reported to be more pronounced than that of calcium (Bryla et al., 2021; Wu et al., 2023). As the electrical conductivity (EC) of any system increases with the increasing abundance of neutral salt species, it is the most widely used determinant for assessing the degree of soil salinity. Sodic soils, on the other hand, are formed by the excessive accumulation of sodium ions in the soil exchange complex. These are often characterized by high pH and dispersibility (and consequent poor soil transmissibility). Carbonates and bicarbonates of sodium play major role in the development of soil sodicity. Exchangeable Sodium Percentage (ESP) and Sodium Adsorption Ratio (SAR) are the most useful parameters to assess the hazard of sodium in soil and soil solution, respectively. Classification of salt affected soils based on soil pH, Electrical Conductivity (EC), ESP, and SAR is given in **Table 1**.

**Table 1:** Different types of salt affected soil.

Type of Soil	pH of Soil	Soil EC (dS m^-1^)	Soil ESP	Soil SAR
Saline	< 8.5	> 4.0	< 15	<13
Sodic	>8.5	Variable	> 15	>13
Saline-Sodic	> 8.5	> 4.0	Variable	>13

Source: Modified from Stavi et al. (2021).

There are two types of salinity based upon salt accumulation: dry land salinity and irrigation salinity (Chakraborty et al., 2013; McFarlane et al., 2016; Stavi et al., 2021). Dry land salinity refers to the accumulation of salts in the soil surface of non-irrigated lands. There are three general processes which are associated with dryland salinity: deep drainage, groundwater movement, and groundwater discharge. Dry land salinity may be classified into two categories: Primary, where salinity occurs naturally, and secondary, where salinity is caused by human activities, such as agriculture. Irrigation salinity occurs when rigorous irrigation with groundwater builds up salinity on the surface of soil through repeated salt accumulation, due to the leaching of irrigation water but salts (Chakraborty et al., 2013; McFarlane et al., 2016 and Zaman et al., 2018).

Plants are amenable to the detrimental effects of salinity throughout their life-cycle but are most vulnerable during the germination and seedling stage. The negative effects of salt on plant growth are related to a reduction in the osmotic potential of growing media, specific ion toxicity, and nutrient imbalance (Greenway and Munns, 1980; Askari-Khorasgani et al., 2021 and Lu et al., 2023). The level of salt tolerance in plants is determined by osmotic adjustment and ionic homeostasis (Acosta-Motos et al., 2017). Excess ions in the soil water lower the solute potential (ψ_s_) and thereby the total water potential (ψ_w_) of the soil. To maintain water uptake and turgor under such conditions, plants need to keep their internal water potential (ψ_w_) below that of the soil (Taiz et al., 2015) Osmotic adjustment is mediated by the accumulation of osmolytes *viz*., organic acids, sugars, and amino acids in plant cells under salt stress. Increased accumulation of osmolytes helps plants lower their water potential to facilitate water uptake from saline soils (Acosta-Motos et al., 2017; Ma et al., 2020; Munns et al., 2020; Zhao et al., 2021). Plants need to maintain a balance between the accumulation of sodium (Na^+^) and the loss of potassium (K^+^) from the cell through ion homeostasis to ensure proper cellular functions. A high potassium-to-sodium (K^+^: Na^+^) ratio in tissues serves as an important indicator of higher salt tolerance in plants. Key strategies to maintain a higher K^+^:Na^+^ involve selective ion uptake and transport mechanisms that allow plants to either exclude or compartmentalize excess Na^+^, and retain K^+^ (Tester and Davenport, 2003; Hasanuzzaman et al., 2018; Ketehouli et al., 2019; Kumari et al., 2021). Therefore, a clear understanding of the mechanisms of salt tolerance at physiological, biochemical, and molecular levels, underlying genetics, and chromosomal regions associated with salt tolerance helps in the identification and further exploitation of tolerant genotypes. The impact of salinity on plants, their stress tolerance mechanisms, and the deployment of modern genomic and breeding approaches to understanding the genetics and mitigation of salt stress are explored in this review article.

## 2 Current global scenario of soil salinity

**Figure 1:**
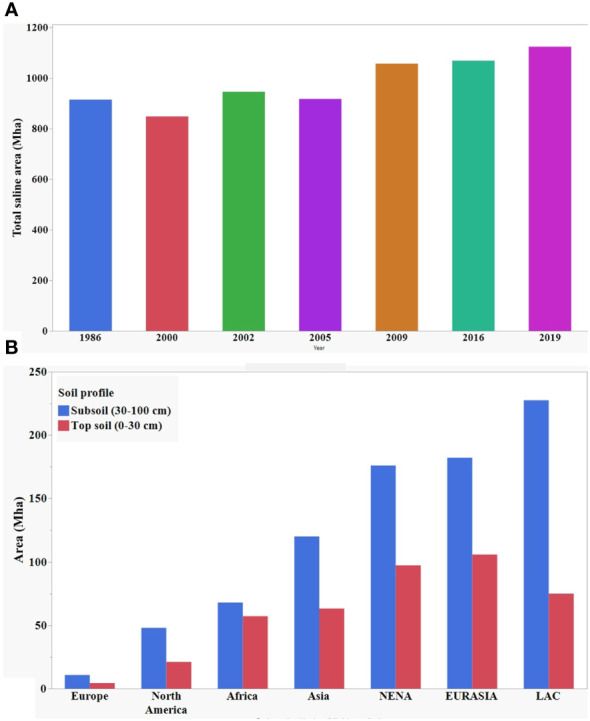
**(A)** Year wise distribution of global total saline area. **(B)** Global distribution of salt affected area at topsoil and subsoil. Data source: Ivushkin et al. (2019); Hossain (2019), and FAO (2021).

The increase in soil salinity poses a serious threat to agriculture production worldwide. Here, **Figure 1A** shows the gradual increase of total salt affected land area over thirty years. Globally, more than one billion hectares of land is affected in more than 100 countries and these numbers are constantly growing (Szabolcs, 1989; Squires and Glenn, 2004; Abbas et al., 2013; FAO and ITPS, 2015; Hossain, 2019; Ivushkin et al., 2019). According to Hossain (2019), about 1125 million hectares of land are affected by salinity at the present time, of which 76 million are affected by human-induced salinization and sodification, and 1.5 million hectares become unsuitable for agricultural production each year due to rising salinity levels. Recently published data by Food and Agricultural Organization (FAO) in 2021 showed global distribution of saline land area at topsoil (0-30 cm) and subsoil (30-100 cm) profile (**Figure 1B**). The salinization of the soil will result in the loss of 50% of cultivable lands by 2050 if it continues increasing at the present rate (Kumar and Sharma, 2020).

## 3 Brief account of physiological and biochemical alterations under salinity stress

### 3.1 Effect on germination and seedling stage

**Figure 2:**
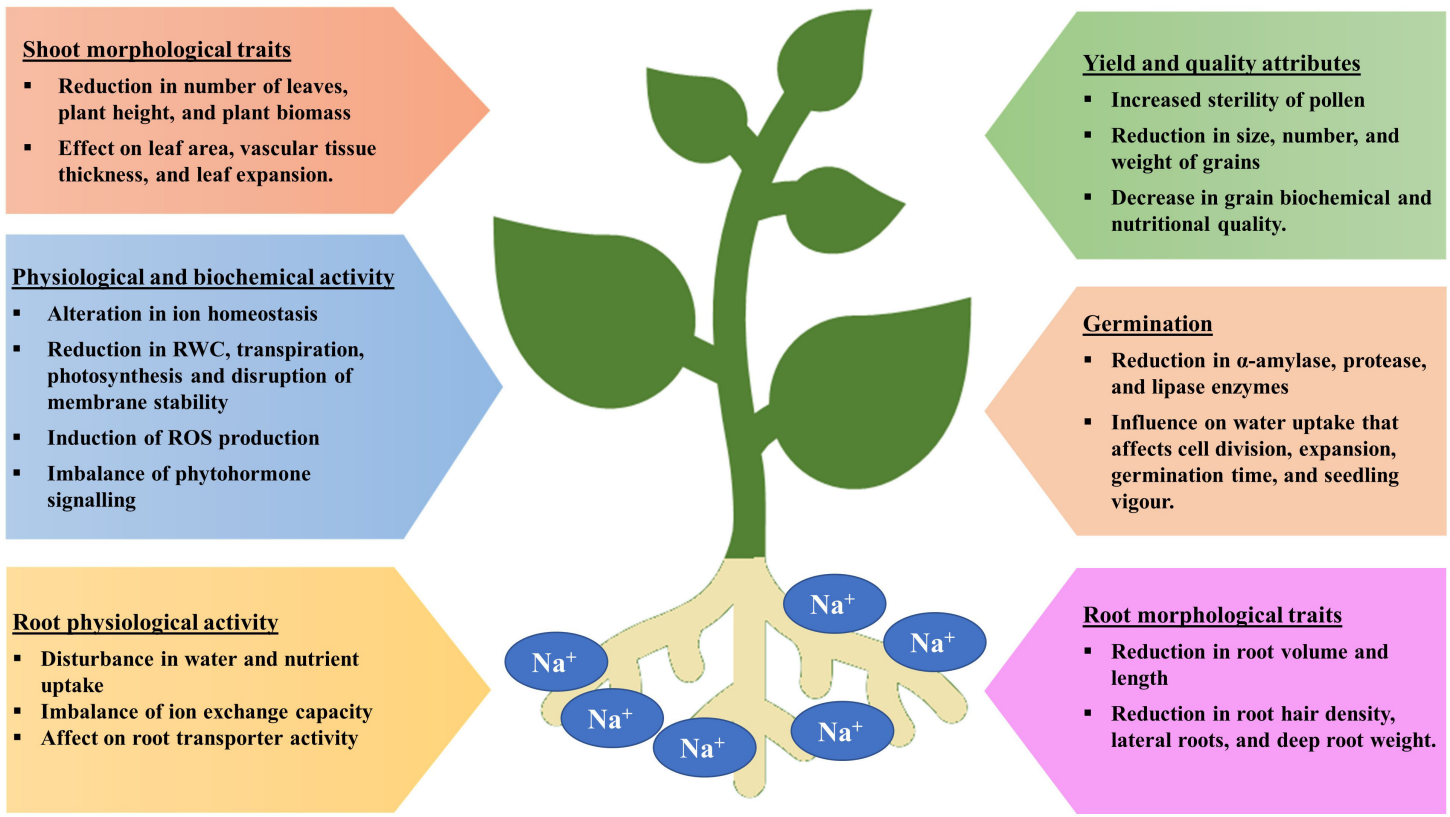
Detrimental effects of salinity stress on various plant parts at different growth stages.

Seed germination is a multi-stage developmental process that is influenced by both internal and external factors. Impact on seed germination under salinity stress may be attributed to the delayed absorption of water and a decline in the activity of α-amylase, an enzyme involved in starch hydrolysis. Salinity lowers the soil osmotic potential relative to the internal osmotic potential of seed, which inhibits the absorption of water during seed imbibition (**Figure 2**) (Munns, 2002 and Munns et al., 2020). As a consequence, the seed germination rate is reduced and the seed germination period is delayed. Even after germination, salinity may also have detrimental effect on embryo viability due to the excess accumulation of Na^+^ and Cl^-^ ions (Daszkowska-Golec, 2011; EL Sabagh et al., 2021). Furthermore, salt stress increases the generation of reactive oxygen species (ROS) and oxidative damages, which disrupt different macromolecules. Therefore, a decrease in α-amylase activity results in a significant reduction in the transfer of sugars, which restricts the embryo’s growth and development. (Hasanuzzaman et al., 2021).

Under normal conditions, seed germination occurs in three phases. Dry seeds absorb water rapidly (imbibition) during Phase I of germination. In phase II, metabolic processes are reactivated and water uptake is limited. Phase III is the post-germination phase, during which continuous water uptake occurs until full germination. Phase I osmotic stress and phase II ionic stress are generally attributed to inhibiting seed germination or delaying germination under salinity stress (Yadav et al., 2011). However, even after seed germination, the development of embryonic organs, seedling growth, and vigor are affected at phase III by both ionic and osmotic stress (Wahid et al., 2014; El-Hendawy et al., 2019).

Salinity impose deleterious effects on seed germination by decreasing gibberellic acid levels, increasing abscisic acid levels, altering membrane permeability, and reducing water absorption in seeds. (Lee and Luan, 2012). Germination of seeds occurs when it is catalyzed by hydrolytic enzymes such as α-amylase which subsequently breaks down the starch stored in the endosperm into metabolizable sugars that provide energy to the growing embryo and radicle. (Weiss and Ori, 2007). For most crops, although seed may be germinated under a certain limit of salinity, it significantly delays the seed germination. The major causes of a delay in germination time may include a delay in water intake and a decrease in α-amylase activity with an increase in NaCl content (Kaneko et al., 2002). It has been noted that the salt-sensitive genotypes exhibit a greater decline in α-amylase activity than the salt-tolerant genotypes. This decrease in α-amylase activity has a substantial impact on the translocation of sugars, which is crucial for the development of the embryo (Uçarlı, 2020).

### 3.2 Ionic imbalance and salinity mediated nutritional deficiencies

**Figure 3:**
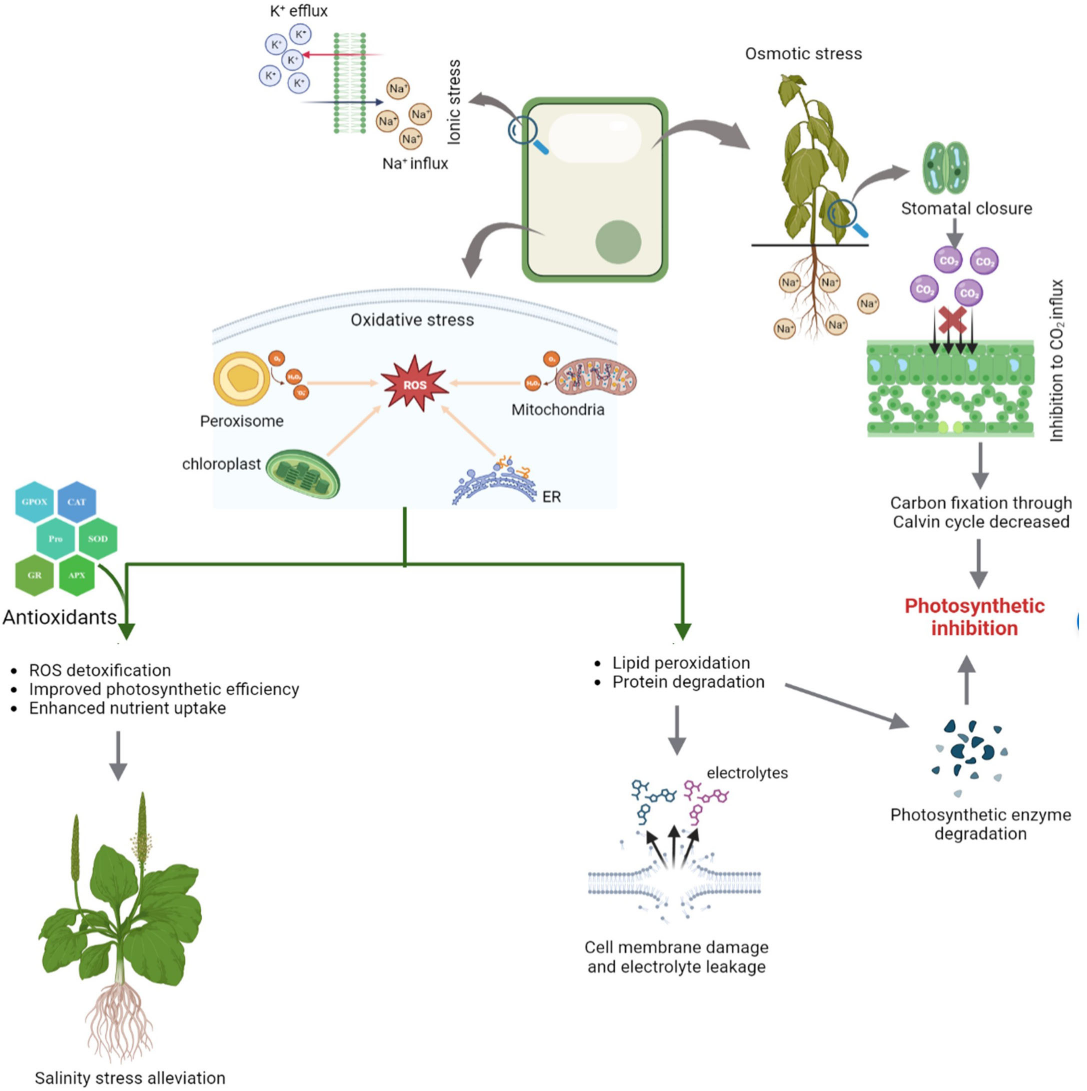
Different physiological alterations in plants under salinity and role of antioxidants in stress alleviation.

It is also important to note that some ions can also act as plant nutrients, such as K^+^ and SO_4_^2-^, while Na^+^ is not considered to be an essential plant nutrient. Thus, soil salinity is often measured in terms of Na^+^ and Cl^−^ (Stavi et al., 2021). Plants are affected by salinity in three ways. Because of its low osmotic potential, salt makes it difficult for plants to extract water from the soil, subjecting plants to osmotic stress, which limits growth and reduces yield. The Na^+^ and Cl^-^ ions when absorbed and accumulated into the tissues by plants at excess concentrations from soil cause cytotoxicity which eventually result in leaf firing, reduced growth, and finally plant death. Moreover, high levels of Na^+^ decrease the availability of other ions such as K^+^, Ca^2+^, and Mg^2+^ due to the cation competition, which can lead to nutrient deficiencies (Munns and Tester, 2008; Atta et al., 2019; Yildiz et al., 2020; Atta et al., 2021). During salinity, the plant takes up more Na^+^ than K^+^ as the amount of Na^+^ in the growth medium increases, increasing K^+^ efflux from the cell and raising the Na/K ratio (**Figure 3**) (Parvin et al., 2016; Rahman et al., 2016). During salt stress, excessive Na^+^ influx encourages ion channel disruption, nutrient replacement and membrane depolarization, leading to abnormalities in nutrient uptake and assimilation (Shabala et al., 2007; Zhao et al., 2007; Nahar et al., 2016; Gaikwad et al., 2022). According to a study in rice by Farooq et al. (2022), the concentrations of all measured nutrients (Fe, K, Mn, Mg, P, and Zn) in the roots and shoots declined under salinity stress. Salinity stress significantly reduces the root surface area by lowering root hair density and root hair length which are directly proportional to nutrient uptake (Robin et al., 2016 and Arif et al., 2019). Essential elements including Ca^2+^, Mg^2+^, Fe^2+^, and Zn^2+^, which are impacted by salt stress, are required for normal root growth. Therefore, the decrease in root growth can further affect the intake of Ca^2+^, Mg^2+^, Fe^2+^, and Zn^2+^ (EL Sabagh et al., 2021; Robin et al., 2016). According to certain studies, corn shoots under salinity stress had lower Mn^2+^ levels (Rahman et al., 1993). Nahar et al. (2016) reported, salt stress decreased the amounts of Ca^2+^, Mg^2+^, and Zn^2+^ in the leaves of mung bean seedlings.

### 3.3 Oxidative stress caused by salinity

Along with its direct effects on plants, salinity frequently results in an excessive build-up of reactive oxygen species (ROS), which can interact with other essential components of plant cells and cause oxidative damage in plants, such as DNA damage, lipid peroxidation, enzyme inactivation, protein oxidation, hormone and nutritional imbalances (Hasanuzzaman et al., 2021). ROS are primarily produced in the chloroplasts, mitochondria, endoplasmic reticulum, cytosol, and peroxisome (**Figure 3**). Stomatal closure caused by salt stress can decrease the amount of available carbon dioxide in the leaves and thus, induces photosynthetic inhibition (Kamran et al., 2020). The light reactions in the chloroplast have a pivotal role in the production of a majority of ROS such as superoxide (O_2_^•-^), hydrogen peroxide (H_2_O_2_), hydroxyl radical (OH^•^), and singlet oxygen (^1^O_2_) (Parida and Das, 2005; Ahmad and Sharma, 2008; Ahmad et al., 2010a; Khorobrykh et al., 2020).

The complex nature of salt stress coupled with water deficit have a variety of detrimental effects on plant metabolic process which in turn produce ROS that affect plant systems. Previous studies have shown oxidative damage caused by salt in many crops, *viz.* in *Oryza sativa* (Xu et al., 2011; Rahman et al., 2016), *Zea mays* (Khodarahmpour et al., 2012), *Vigna radiata* (Nahar et al., 2016) and *Solanum lycopersicum* (Kaveh et al., 2011). They also reported reduced ascorbate (AsA) to dehydroascorbate (DHA) ratio, superoxide dismutase (SOD) and catalase (CAT) activity, along with an increase of methylglyoxal (MG) content, which collectively contribute to oxidative damage. Greater levels of H_2_O_2_, as well as higher MDA and overproduced MG, were also reported in rice seedlings under salt stress, where Lipoxygenase (LOX) and SOD activity increased while CAT activity decreased (Rahman et al., 2016).

### 3.4 Effect on yield and yield components

There have been numerous studies demonstrating that abiotic stresses, like salinity, cause significant yield losses in major crops during the reproductive stage (Kalhoro et al., 2016; Ehtaiwesh and Rashed, 2020). During the reproductive stage, Na^+^ is excluded from leaf blades by the class I high-affinity K^+^ transporter (HKT) family, affecting sodium ion homeostasis under salinity stress (Suzuki et al., 2016). It was also found that grain dry matter and the K^+^/Na^+^ ratio was significantly correlated with grain filling rate and duration under salt stress (Poustini and Siosemardeh, 2004).

Changes in water relations, transpiration, nutritional imbalances, stomatal conductance, and oxidative damage due to salt stress all contribute to a drop in yield. By altering morpho-physiological and biochemical processes, salinity reduces agricultural yield and production (Kaveh et al., 2011). Additionally, it slows down photosynthetic activity, biomass accumulation, and source-sink activity, which has a negative impact on yield response variables and speeds up the senescence of reproductive organs (Khataar et al., 2018). Similar to this, throughout the reproductive phase, changes in water potential result in decreased flag leaf and vascular tissue thickness, mesophyll cell size, cell elongation, and epidermal cell size, which affect the leaf area, flag leaf turgidity, assimilate synthesis, and ultimately yield potential (Farouk, 2011). However, Ashraf and Ashraf (2016) hypothesized that changes in such biochemical and physiological characteristic are stage-specific and related to yield attributes. Salinity, for instance, reduces grain output by 39.1, 24.3, and 13.4%, respectively, at various stages of wheat like anthesis, early booting, and mid grain filling (Ashraf and Ashraf, 2016; Sabagh et al., 2021). Nadeem et al. (2020) found that salinity had a detrimental effect on wheat crop yield (yield, test weight and grain length), nutritional quality features (gluten content, fiber, fat, ash and moisture), and mineral element content (Mg, P, Ca, Zn, K and Fe). At 8 dSm^−1^, yield per plant in *Brassica oleracea* var. *capitata* can drop by up to 62% (Parvin et al., 2017). Salinity has a considerable impact on crop reproductive responses and yield contributing qualities, resulting in yield loss (Parvin et al., 2015a). Various crop species, including moong bean (Nahar and Hasanuzzaman, 2009), *B. oleracea* var. *capitata* (Parvin et al., 2017), tomato (Parvin et al., 2015a) and *B. oleracea* var. *italica* (Parvin and Haque, 2017), showed reduced yield component and yield under salt stress. **Table 2** illustrates the extent to which salinity stress reduces crop yields.

**Table 2:** Yield reduction due to salinity stress on different crops.

Crops	Yield reduction (%)	References
Rice (*Oryza sativa*)	30-50	Eynard et al. (2005); Hoang et al. (2015)
Barley (*Hordeum vulgare*)	10-60	Abd El-Monem et al. (2013)
Wheat (*Triticum aestivum*)	10-50	Panta et al. (2014); Zorb et al. (2018); Yadav et al. (2020)
Bajra (*Pennisetum glaucam*)	5-25	Yadav et al. (2020)
Sorghum (*Sorghum bicolor*)	15-50	Daniells et al. (2001); Kenneth and Neeltje (2002)
Maize (*Zea mays*)	50-60	Zorb et al. (2018)
Chickpea (*Cicer arietinum*)	10-30	Atieno et al. (2017)
Brassica sp. (*Brassica napus & B. juncea*)	30-70	Pavlović et al. (2019); Singh et al. (2021a)
Sugarcane (*Saccharum officinarum*)	5-60	Plaut et al. (2000); Rao et al. (2015); Kumar et al. (2023)
Groundnut (*Arachis hypogaea*)	25-50	Otitoloju (2014); Zorb et al. (2018)
Cotton (*Gossypium* sp.)	10-20	Zorb et al. (2018)

## 4 Plant adaptations to salinity stress and mechanisms

In high salinity soils, plants develop a range of physiological and biochemical adaptations. In addition to ion homeostasis and compartmentalization, the principal responses include biosynthesis of compatible solutes, osmo-protectants, antioxidant compounds, polyamines, and nitrous oxide, as well as regulation of phyto-hormones. The following sections discuss recent advances in elucidating these mechanisms.

### 4.1 Ion uptake, transport, compartmentalization, and homeostasis

Plant organs are unable to function when their tissues or cells contain excessive Na^+^ or Cl^–^ concentrations. Increased Na^+^ concentration usually results in a decrease in K^+^, and it may be critical for tolerance to maintain cytosolic K^+^ levels at an acceptable level or to maintain ‘homeostasis’ (Shabala and Pottosin, 2014). The toxic effect of Na^+^ may also be a result of its competition with K^+^ for enzymes that require K^+^, such that the ratio of cytoplasmic Na^+^ to K^+^ may be more important than the concentration of Na^+^ itself (Shabala and Cuin, 2008).

Neither glycophytes nor halophytes can tolerate high salt concentrations in their cytoplasm; however halophytes have developed mechanisms to sequester these ions. Na^+^ ion exclusion or compartmentalization’s are essential for normal plant growth under salinity stress (Serrano et al., 1999; Hasegawa, 2013). In saline soils, the most abundant salt form is NaCl, therefore most research has been on how Na^+^ ions are transported and compartmentalized in cells. During salinity stress, cell membranes and their associated components regulate ion uptake and transport within the cytosol (Sairam and Tyagi, 2004). This results in either excess salt being transported to the vacuole or sequestered in older tissues that eventually senesce, thereby protecting plants from salt stress (Reddy et al., 1992; Zhu, 2003). Carrier proteins, channel proteins, antiporters, and symporters all take part in ion transport. Some well characterized transporters include Na^+^ transporter *AtHKT1:1* (Rus et al., 2004), Na^+^/H^+^ antiporter *AtNHX1* (Apse et al., 1999), K^+^/Na^+^ symporter *TaHKT1* (Schachtman and Schroeder, 1994) and Na^+^/H^+^ antiporter *SOS1* (Zhu, 2003). These antiporters facilitate the compartmentalization of excess ions through the movement of Na^+^ ions into vacuoles from the cytoplasm (Gupta and Huang, 2014; Segami et al., 2018). After Na^+^ ions enter the cytoplasm in excess, antiporters transport them into vacuoles. Vacuolar membrane H^+^ pumps exist in two forms: the vacuolar type H^+^-ATPase (V-ATPase) and the vacuolar pyro-phosphatase (V-PPase); V-ATPase being the predominant form which generate motive forces across the vacuolar membrane. The ability of the plant to survive under high salinity depends to a great extent on its V-ATPase activity (Dietz et al., 2001).

### 4.2 Osmoprotection via compatible solutes

Compatible solutes, also called osmolytes are uncharged, polar, and soluble molecules which usually do not interfere with the cellular metabolism even at high concentrations. Organic osmolytes are synthesized and accumulated in varying amounts amongst different plant species to adjust osmotic potentials and protect cells. They are most commonly proline (Ahmad et al., 2010b; Hossain et al., 2011; Nounjan et al., 2012; Tahir et al., 2012), glycine betaine (Khan et al., 2000; Wang and Nii, 2000), sugars (Bohnert et al., 1995; Kerepesi and Galiba, 1995), and polyols (Ashraf and Foolad, 2007; Saxena et al., 2013). The amino acid proline has been found in diverse taxonomical groups of plants (Saxena et al., 2013), while accumulations of beta alanine betaine have been observed in plants of the Plumbaginaceae family which are glycophytes (Hanson et al., 1994 Matysik et al., 2002; Ben Ahmed et al., 2010). Under salinity stress, the concentrations of cysteine, arginine, and methionine, which represent about 55% of total free amino acids, decreased, whereas proline concentration increased (El-Shintinawy and El-Shourbagy, 2001). Additionally, proline accumulated in the intracellular space during salt stress also serves as an organic nitrogen reserve during stress recovery. In salt-stressed plants, sugars like glucose, fructose, fructans, and trehalose are also accumulated (Parida et al., 2004). These carbohydrates facilitate stress mitigation via, osmoprotection, and neutralization of reactive oxygen species. Many plant species have been reported to increase their levels of reducing and non-reducing sugars (sucrose and fructans) when they are under salinity stress (Kerepesi and Galiba, 1995). Trehalose accumulation is not only a carbohydrate reserve but also a protective mechanism against several stresses including salinity (Ahmad et al., 2013). A reduction in sucrose content was observed in tomato (*Solanum lycopersicum*) when exposed to salinity; due to an increase in saccharophosphate synthase activity (Gao et al., 1998). Different rice genotypes have been reported to both increase and decrease their sugar content under salinity stress (Alamgir and Yousuf Ali, 1999). Compatible solutes stabilize cellular structures and enzymes, act as metabolic signals, and scavenge ROS. Osmoprotection through compatible solutes is thus an important mechanism for plants to mitigate the negative effects of salinity stress.

### 4.3 Antioxidant regulation

Abiotic stresses including salinity result in electron overflow, deregulation, and even disruption of electron transport chains (ETCs) in chloroplasts and mitochondria. ROS produced under salinity stress are scavenged by enzymatic oxidants (SOD- Superoxide dismutase, APX- Ascorbate peroxidise, GPX- glutathione reductase, CAT- catalase, PPO- polyphenol oxidase, MDHA- monodehydroascorbate, MDHAR- monodehydroascorbate reductase etc.) as well as non-enzymatic antioxidants (reduced glutathione, flavanoids, phenolics, α-tocopherol, alkaloids etc.) which protect the plants from oxidative damage (**Figure 3**) (Hasanuzzaman et al., 2021). Among these, Ascorbate peroxidase (APX) and glutathione reductase (GR) are important antioxidant enzymes positively related to salt tolerance (Asada, 1999; Gupta et al., 2005). At lower concentration, ROS act as signaling molecules which initiates complex cascade of pathways and interactions (Golldack et al., 2014). Of these, MAPK (mitogen-activated protein kinase) and salt overly sensitive (SOS) signaling pathway cascades are important mediators of osmotic, ionic and ROS homoeostasis (Huang et al., 2019). ROS signals, through these pathways, trigger antioxidant defense mechanisms and scavenging of ROS (Kurusu et al., 2015).

### 4.4 Role of polyamines

Polyamines (PA) are cationic, aliphatic, and low molecular weight molecules which play a variety of roles in normal growth and development, including cell proliferation, morphogenesis, and growth of flowers and fruits. Plants show higher tolerance to stresses when polyamine levels rise (Yang et al., 2007; Groppa and Benavides, 2008; Kovács et al., 2010; Gupta et al., 2013a; Gupta et al., 2013b). Under salinity stress, polyamines contribute to cellular responses by modulating ROS homeostasis (Takahashi and Kakehi, 2010). Some research has suggested exogenous application of polyamines helps to alleviate salinity stress (Minocha et al., 2014; Rathinapriya et al., 2020) whereas it has also been suggested that catabolism products of polyamines (like H_2_O_2_) limit the ability of plants to tolerate stress (Saha et al., 2015). Further investigations on the role of polyamines are needed in this regard before conclusive remarks can be made.

### 4.5 Roles of nitric oxide

The molecule Nitric Oxide (NO) is a small volatile gas that plays an important role in many important plant processes, including regulating root growth, respiration, stomata closure, flowering, cell death, seed germination, and stress responses (Crawford, 2006; Besson-Bard et al., 2008; Zhao et al., 2009). Many redox-regulated genes are induced by NO directly or indirectly. By interacting with lipid radicals, NO prevents lipid oxidation, scavenging superoxide radicals and forming peroxynitrite that can be neutralized by other cellular processes. Additionally, it activates antioxidant enzymes (SOD, CAT, GPX, APX, and GR) (Bajgu, 2014). There is evidence that exogenous NO application can mitigate stress but the effects are dependent on NO concentration (Hossain et al., 2010; Sung and Hong, 2010; Xiong et al., 2010). NO has been demonstrated to mediate salt stress tolerance in plants (Wang et al., 2012) by counteracting germination inhibition (Zheng et al., 2009), negating inhibition of growth (Ageeva-Kieferle et al., 2019) and by its role in ion-homeostasis (Zhao et al., 2004).

### 4.6 Hormonal regulation of salinity tolerance

Among the well characterized plant hormones, abscisic acid (ABA), salicylic acid, jasmonic acid, and ethylene are considered as stress response hormones (Verma et al., 2016). Many studies have elucidated that these phytohormones have sophisticated roles in plant systems and their action is growth stage, tissue and environment specific (Ku et al., 2018). As a consequence of osmotic stress and water deficit, salt stress increases ABA production in vascular tissues and its distribution in roots and shoots (Popova et al., 1995; Jeschke et al., 1997; Cramer and Quarrie, 2002; Kang et al., 2005; Cabot et al., 2009). There is evidence that the positive association between ABA accumulation and salinity tolerance is at least partially due to the accumulation of potassium, calcium, and compatible solutes, such as proline and sugars, within the vacuoles of roots, which counteract Na^+^ and Cl^-^ uptake (Golldack et al., 2014). In rice seedlings, the level of endogenous salicylic acid increased under salinity stress (Sawada et al., 2006). Furthermore, salinity can be mitigated by brassinosteroids (Krishna, 2003; Ashraf et al., 2010; El-Mashad and Mohamed, 2012). As a result of brassinosteroid application, the antioxidant enzymes SOD, POX, APX, and GPX were increased and non-enzymatic antioxidant compounds (tocopherol, ascorbate, and reduced glutathione) were accumulated (El-Mashad and Mohamed, 2012). Studies on *Arabidopsis* provide most of our information about hormone-mediated salt stress tolerance, but rice and maize might not conserve the same regulatory mechanisms (Yu et al., 2020). In order to guide their application and translation into agricultural production, further research on hormones in crop plants is required in order to elucidate their role in salt tolerance.

## 5 Phenomics

Irrespective of the breeding approach adopted, be it conventional or modern, precise phenotyping stands as the pivotal determinant of success in any breeding program. The expeditious advancement of crop breeding programs heavily rely on the adoption of sensor-based automated high throughput phenotyping (HTP). Phenomics aids in getting an extra genetic gain when it is used along with genomic studies by increasing selection intensity and accuracy (Lozada and Carter, 2020; Sandhu et al., 2021b). Recent advances in phenomics facilitate acquiring robust, non-invasive, high throughput phenotyping that quantify different morphological and physiological parameters of plants. In case of osmotic stress (due to salt stress), plants try to reduce transpiration loss by reducing stomatal conductance. Low transpiration leads to increase leaf surface temperature. Thermal Infra-Red (IR) imaging can sense the change in leaf surface temperature more precisely between stressed and non-stressed plants under increasing stress level. Sirault et al. (2009) optimized a high throughput screening protocol to quantify osmotic stress response in barley, grown in a range of salt concentrations, using IR thermography based on visualizing the leaf temperature differences due to the variation in stomatal conductance. In another study, plant temperature captured by thermal images showed negative correlation with stomatal conductance and relative water content and no relation with photosynthetic quantum yield in rice under salt stress environment Siddiqui et al., 2014).

Another tool used for extracting information on plant structural and physiological trait is optical imaging. Among these, Red-Green-Blue (RGB) imaging and hyper spectral imaging (HSI) are two most widely used and promising phenotyping approaches. A number of plant morphological changes like change in plant canopy area, compactness, leaf colour are associated with salt stress. Dissanayake et al. (2020) screened 276 accessions of lentil for salt tolerance using RGB imaging where the discrimination between tolerant and susceptible genotypes were done based on projected shoot area, leaf colour, height, convex hull area, and compactness. In a different study, genetic variation of 245 diverse chickpea accessions under elevated salt was assessed using image-based phenotyping (projected shoot area, senescence) and analytic measurements (leaf Na^+^ and K^+^ content, biomass, pod number, and 100-seed weight) where pod and seed numbers found to be the most important traits under consideration for breeding chickpea with improved salinity tolerance (Atieno et al., 2017).

Chlorophyll fluorescence (ChlF) is a potential indicator of stress induced changes in photosynthetic mechanisms (PSII activity, photochemical and non-photochemical quenching, electron transport rate) of plants. Tissue tolerance to Na^+^ ion toxicity in rice was determined by steady state ChlF imaging. The fluorescent images captured can measure chlorotic or necrotic area and thus can separate healthy plant parts from senescent parts (Hairmansis et al., 2014). Similar studies have been conducted in soybean (Kim et al., 2014), sunflower (Neto et al., 2011), acer (Percival et al., 2003), and grape (Dunlevy et al., 2022) for the development and optimization of salt tolerance screening protocol. However, steady state ChlF imaging cannot provide insight into the photosynthetic activity of plants, so kinetic ChlF has been introduced that can quantify the photosynthetic performance under stress condition. Awlia et al. (2016) studied rosette area, rosette colour and photosynthetic performance of *Arabidopsis thaliana* under salt stress using kinetic ChlF imaging in conjunction with RGB imaging. Fluorescence imaging revealed that non-photochemical quenching and quantum yield are two important traits related to salt stress that induced Arabidopsis plant performance for early and late phase salt stress, respectively. In another study Tsai et al. (2019) reported that in a salt stress sensitive rice variety, the maximum quantum efficiency of PSII was much lower than a tolerant one after NaCl treatment. Al-Tamimi et al. (2016) used RGB based phenotyping to conduct a genome wide association study (explained later in section 7) in rice and identified loci related to transpiration use efficiency. Similarly, in a separate study two novel candidate genes, *BnCKX5* and *BnERF3*, linked to salt stress in *Brassica napus* were discovered by high-throughput phenotyping-based QTL mapping. (Zhang et al., 2022a). Consequently, to achieve a better grasp of the genetic mechanisms governing stress tolerance and to accelerate breeding endeavors targeting these traits, it is imperative to prioritize the incorporation of phenomic tools within breeding programs.

## 6 Mapping of salinity tolerance genes

Use of existing genetic variation within or among the species or the creation of variations through mutation or other genetic engineering approaches is a prerequisite to crop improvement. Efficient utilization of such tolerant genotypes as donors in crop improvement programs either through conventional breeding or genetic engineering necessitates unravelling the underlying genetics of salt tolerance. Incorporation of tolerance genes from tolerant lines to agronomically superior but susceptible lines through Marker Assisted Selection (MAS), is a widely followed crop improvement scheme. Mapping those genes is the elementary and crucial step for successful MAS. The genomic regions that contain genes which influence the expression of quantitative characters are referred to as Quantitative Trait Loci (QTL) (Tanksley et al., 1996; Collard et al., 2005). Bi-parental mapping of QTL has been reported to provide valuable information for further map-based cloning of salt tolerance genes and MAS in many economically important crops like rice, maize, pearl millet etc. (Sharma et al., 2011; Tiwari et al., 2016; Luo et al., 2017).

The major constraints in research progress for salinity tolerance are attributed to the polygenic inheritance pattern and significant influence by genotype and environment interactions (Zhu, 2000; Singh et al., 2007; Wang et al., 2014). The basic principle behind the QTL analysis aims to detect an association between phenotype and genotype within the population (Collard et al., 2005). However, mapping of salt tolerance is highly influenced by screening protocol and phenotyping accuracy of the traits, size of the mapping population, linkage between markers and QTL, and parental specificity. Phenotypic response of the plants under salinity stress depends on the age of the plant, duration and level of the salt treatment (Singh et al., 2007). Therefore, a reliable mapping of QTL necessitates accurate phenotyping as well as genotyping of the population.

Major crop-specific achievements in QTL mapping for salinity tolerance through bi-parental populations in recent years, valuable stress indicative parameters, and the importance of crop growth stage while screening are discussed in the following section.

### 6.1 Rice

Several mapping studies in regard to salinity tolerance have been conducted on rice. Although the reproductive stage is considered more critical than the seedling stage as it directly affects grain yield, major reports on QTL are limited to the seedling stage to avoid cumbersome phenotyping efforts (Singh et al., 2021b). *Saltol* is a widely known QTL in rice at the seedling stage, which has been reported by several groups of researchers (Bonilla et al., 2002; Niones et al., 2006; Thomson et al., 2010). Mapping of an *indica* Recombinant Inbred Lines (RIL) population identified six QTL at the seedling stage distributed on chromosomes 1 and 4 (Dahanayaka et al., 2017). Puram et al. (2018) used a set of introgressed Lines (IL) from donor parent ‘Nona Bokra’ and identified 18 QTL for salt tolerance indices. This study suggested shoot Na^+^ exclusion, Na^+^: K^+^ homeostasis, and compartmentation of Na^+^ as probable salt tolerance mechanisms in ‘Nona Bokra’. Another QTL mapping study conducted on an F_2_ population at the reproductive stage, detected sixteen QTL related to salinity stress on four linkage groups (Hossain et al., 2015).

### 6.2 Wheat

QTL mapping on 350 RILs identified 90 stable QTL for 15 traits with a genome-wide distribution except for chromosomes 4D, 6B, and 7D. Out of four QTL clusters that were located on chromosomes 2D, 3D, 4B, and 6A, eight notable QTL were validated in a collected natural population. Among them, one QTL was found to be associated with the dwarfing gene *Rht-B1* (*Rht*- Reduced height) which is responsible for reduced plant height and increased seed yield. Additionally, three Kompetitive Allele-Specific PCR (KASP) markers derived from SNPs were successfully designed for three QTL clusters (Luo et al., 2021b). Another study in a RIL population comprising of 254 individuals revealed a total of 158 stable additive QTL for 27 morpho-physiological traits distributed over all the wheat chromosomes except 3A and 4D. 78 out of the 158 QTL, were mapped in nine QTL clusters and seven QTL were validated in two unique populations to check the reliability and potential use in MAS, leading to the development of KASP markers closely linked to stable QTL (Luo et al., 2021a). Rezaei et al. (2021) detected a total of 61 main effect QTL distributed over 15 chromosomes from a study of 186 F_10_ RILs during the germination and early-seedling stages. Two major QTLs for primary-leaf fresh weight and coleoptile fresh weight were detected on chromosomes 5 and 2, respectively (Rezaei et al., 2021). Asif et al. (2020) identified six QTL for salt tolerance traits: sodium accumulation, chloride accumulation, K^+^-Na^+^ ratio, and maintenance of shoot growth under salinity in a RIL population of wheat developed from biparental mating of Excalibur × Kukri. GBS data of the mapping population was associated with both non-destructive high-throughput imaging data [projected shoot area (PSA), relative growth rate (calculated from PSA)], and destructive data such as Na^+^, K^+^, and Cl^-^ ion content in the leaves.

### 6.3 Maize

In a salt tolerance study of 209 doubled haploid (DH) lines, 41 QTL out of a total of 61 were found to be associated with salt tolerance for biomass related traits. These salt tolerance-specific QTL clustered on chromosomes 1, 3, 7, and 9, among which 13 major effect QTL on chromosome 1 contributed the most to the phenotypic variance (Luo et al., 2019a). Another study by Luo et al. (2017) on a DH population of 240 individuals at maturity stage revealed a major QTL for plant height on Chromosome 1 under salinity. In addition, the major QTL influencing plant height-based salt tolerance index was also mapped at the same position on Chromosome 1, and two candidate genes related to ion homeostasis were identified within the confidence interval of this QTL. Using 161 F_2:5_ RILs, a field as well as a hydroponic experiment were conducted for QTL analysis (Cui et al., 2015). A total of 29 QTL, clustered on chromosomes 1, 3, and 5, were detected. Among those 14 showed significant QTL by treatment (*Q* × *T*) interaction effects.

### 6.4 Sorghum

In a biparental mapping population of 181 RILs, three traits at the germination stage and nine traits at the seedling stage were analyzed, where a total of 12 QTL [(PVE range of 5.4 to 6.0%) and 29 QTL (PVE range 5.3–21.9%) were identified, respectively. Six major QTL at the seedling stage were identified with the positive effects being majorly from the maternal parent. Further extension of this study at the whole plant growth stages detected a total of 53 QTL for six characters for both salt and control conditions. Out of which, six QTL were declared as major QTL (Wang et al., 2020). In a recent study, using a population of 177 F_3:5_ interspecific RILs, a high-density genetic map was generated covering the 10 *Sorghum* chromosomes with 1991 markers. The genetic map was used to identify 10 salt stress-specific QTL related to plant growth and overall plant health. Four of them that affected plant height, total biomass, and root biomass, were found to colocalize on chromosome 4. These salt-responsive QTL contained genes related to osmotic and ionic tolerance along with many aquaporins (Hostetler et al., 2021).

### 6.5 Chickpea

In a recent study in both glasshouse and field conditions, 200 RILs from a cross between two *Cicer arietinum* varieties Rupali (sensitive) and Genesis836 (tolerant), 42 QTL were detected as having effects on different growth parameters. Among them six major QTL on chromosomes four, five, and six were related to salt tolerance. However, in total, 21 QTL were mapped to two distinct regions on chromosome 4 (Atieno et al., 2021). Soren et al. (2020) screened a set of 201 RILs at the reproductive stage under field conditions developed by crossing two diverse parental lines ICCV 10 (salt-tolerant) and DCP 92-3 (salt-sensitive). An association between genotypic and phenotypic data identified 28 QTL in the population, among which one individual QTL on chromosome 6 related to yield contributed to the maximum phenotypic variance (28.4%). Major QTL associated with yield and yield-related components under salinity stress were found to be clustered on CaLG03 and CaLG06. Candidate genes related to salinity tolerance included histidine kinase, Ca-dependent protein kinases, antiporter genes, and transcription factors such as WRKY and MYB.

### 6.6 Soybean

An F_9_ generation RIL population (salt-sensitive cultivar, Cheongja 3 × salt-tolerant landrace, IT162669) consisting of 174 individuals, was screened for salt tolerance at the vegetative stage (Cho et al., 2021). Phenotypic data taken after two weeks of salt stress included major stress-indicative physiological traits such as vegetative damage and Na^+^-K^+^ ion contents. Two novel major QTL on chromosomes 6 and 10 were identified as related to ionic stress and other major physiological parameters, respectively under salinity. Analyses of differential gene expression patterns between parents and functional annotation revealed two potential candidate genes in *qST6* and six in *qST10*, which included a phosphoenolpyruvate carboxylase and an ethylene response factor. Do et al. (2018) developed a population of 132 F_2:3_ (moderately sensitive cultivar Williams 82 × tolerant cultivar Fiskeby III (PI 438471)) and identified major chromosomal loci related to salt stress. Plants were phenotyped with vegetative parameters and ion contents after two weeks of salt stress. A major QTL derived from Fiskeby III located on Chr-03 was found to be significantly associated with leaf scorch, chlorophyll content ratio, and sodium and chloride ion contents. Additionally, another allele related to leaf sodium content was detected and mapped on Chr-13.

### 6.7 Medicago truncatula

In a study on the model legume crop *Medicago truncatula*, a set of 133 RILs (Jemalong A17 (JA17) × F83005.5 (F83)) were screened for salt tolerance (Arraouadi et al., 2012). Dry biomass and accumulation of Na^+^ and K^+^ ion in roots, stems, and leaves were considered for the phenotyping of the plants. Out of 13 QTL spanning eight linkage groups, six QTL in control, two in salt, and five for salt sensitivity index were mapped. Most of the QTL were found to be clustered on Chr-1, however, no QTL were detected on Chr-5 and 6. Identification of non-overlapping QTL for root and leaf traits suggested the involvement of different genes in ion transportation between roots and leaves.

### 6.8 Cotton

In an SNP-based QTL mapping study, a total of 66 QTL were detected from an F_2_-derived F_3_ population of tolerant cotton line CCRI35 and susceptible Nan Dan (NH) (Diouf et al., 2017). Plants were screened with morpho-physiological parameters at the seedling stage under three salt concentrations. Out of all detected QTL, only 14 (10 from the male parent and four from the female parent) for six traits showed consistency across three salt environments, which accounted for 2.72 to 9.87% of PVE. Five and nine QTL were found to be located in the A_t_ and D_t_ sub-genomes, respectively. Further analysis detected eight clusters that were associated with 12 putative key genes related to salinity.

### 6.9 Zoysiagrass

Guo et al. (2014) conducted a study in an intraspecific F_1_ mapping population comprising of 120 progeny derived from a cross between salt-tolerant accession *Zoysia japonica* Z105 and salt-sensitive accession Z061. Two QTL having a significant impact on leaf firing were detected on chromosome 4. Another major QTL for reduced shoot clipping dry weight was detected on chromosome 5.

Major findings of above-mentioned QTL studies are summarized in the **Table 3**.

**Table 3:** Summary of major QTL detected in different crops in recent years.

Crop	Type of MP	Major QTL detected	Crop stage	Chromosome	PVE (%)	Author
Rice	100 RILs (At354 × Bg352)	*qSSI1*, *qSL1*, *qSNK1*, *qSL4*, *qSNK4*, *qSSI4*	Seedling	1, 1, 1, 4, 4	10.8, 10, 8.9, 15, 11,16	Dahanayaka et al. (2017)
	112 ILs (Cheniere × Nona -Bokra)	*qK3.1*, *qNaK3.1*, *qSHL8.1*, *qDWT8.1*, *qSRI-K9.1*, *qSRI-NaK9.1*	Seedling	3, 3, 8, 8, 9, 9	14.8, 14.6, 14.2, 17.6	Puram et al. (2018)
	F_2_s (Cheriviruppu × PB1)	*qPH1.1*, *qTN7.3*, *qPL7.4*, *qBM8.2*	Reproductive	1, 7, 7, 8	47.1, 12, 35.1, 17.9	Hossain et al. (2015)
Wheat	254 RILs (ZM175 × XY60)	*QRl-2B.1*, *QsK-4B*, *QTrad-2B*, *QTrsa-2B*, *QMrl-2B*	Seedling	2, 4, 2, 2, 2	46.43, 12.87, 23.05, 15.14, 15.2	Luo et al. (2021a)
	350 RILs (ZM175 ×XY60	*QPh-4B*, *QHi-4B*	Whole-plant growth	4, 4	32.43, 22.02	Luo et al. (2021b)
	184 RILs	*QPfw-5B2*, *QCl-2B1*	Germination and early seedling	5, 2	43.99, 20.38	Rezaei et al. (2021)
	128 RILs Excalibur × Kukri	*QNa:K.asl-2B*, *QCRGR.asl-5A, QNa.asl-2A, QG(1-5).asl-5A*	Seedling	2, 5, 2, 5	14.6, 11.2, 10.3, 10.9	Asif et al. (2020)
Maize	209 DH lines (hybrid Xianyu 335)	*qRLS1*, *qFLS1-2*, *qRLR1*, *qFLS1-2*	Seedling	1, 1, 1, 1	63.19, 55.21, 58.35, 55.21	Luo et al. (2019a)
	240 DH lines (PH6WC × PH4CV)	*qSPH1*, *qPHI1*	Matured	1, 1	31.2, 25.94	Luo et al. (2017)
	161 RILs (F63 × F35)	*QFstr1*, *QStr3*, *QSkcs|skcn3^+^*, *QSkns|sknn3* ^+^	Seedling	1, 3, 3, 3	58.33, 24.98, 47.96, 32.54	Cui et al. (2015)
Sorghum	181 RILs (Shihong137 × L-Tian)	*qSH1*, *qSH4*, *qSFW4*, *qTFW4*, *qTFW1*, *qRL10*-*2*	Seedling	1, 4, 4, 4, 1, 10	13.5, 15.6,11.6, 11.5, 21.9, 16	Wang et al. (2014)
	181 RILs (Shihong137 × L-Tian)	*qTB6, qSFW9, qJW9, qBrix2, qBrix10*, *qSTI-Brix9*	Whole-plant growth	6, 9, 9, 2, 10, 9	11.15, 17.7, 14.4, 12.83, 11.58, 15.45	Wang et al. (2020)
	177 RILs (*S. propinquum* × *S. bicolor*)	*qHT45_4.STI*, *qTB45_4.STI*, *qTB45_4.S*, *qRB45_4.ST*	45 DAT	4, 4, 4, 4	9, 13.4, 10.37, 11.4	Hostetler et al. (2021)
Chickpea	200 RILs (Rupali × Genesis836)	*salSYqtl.2, saltolSYqtl.1*, *saltolSNqtl.2*	Reproductive	4, 5, 4	22, 17.9, 28.5	Atieno et al. (2021)
	201 RILs (F_8_) (ICCV 10 × DCP 92-3)	*qSSIYP6.1, SSI_YP, qSSIYP3.1, and qSSI100SW3.1*	Reproductive	6, 3	12.2-28.3, 10, 10.1	Soren et al. (2020)
Soybean	174 RILs (F_9_) (Cheongja-3 × IT162669)	*qST10 (STR, SPAD, FW, DW), qST6 (Na^+^, K^+^:Na^+^)*	Vegetative	10, 6	20.07-24, 14.07-24.38	Cho et al. (2021)
	132 F_2:3_ (Williams-82 × Fiskeby III)	*qLSS, qCCR, qLSC, qLCC, qLSC*	Vegetative	3, 3, 3, 3, 13	48.2, 31.3, 20.6, 58.9, 11.5	Do et al. (2018)
*M*. *truncatula*	133 RILs (F_8_) (JA17 × F83)	*LeaKCsi.1, LeaNaKCsl.1*, *RoNaKCct.1, LeaNaTQct.1*	Vegetative	1, 1, 1, 1	11.7, 9.8, 10, 10.8	Arraouadi et al. (2012)
Cotton	277 F_2:3_ (CCRI35 × NH)	*qEC_A12_110.2*, *qSLW_A06_110*, *qFW_D03_110.2*	Seedling	A12, A06, D03	8.29, 7.91, 9.87	Diouf et al. (2017)
Zoysiagrass	120 F_1_s (*Z. japonica* Z105 × Z061	*qLF-1*, *qLF-2*, *qSCW-1*	30 DAT	4, 4, 5	13.1, 29.7, 65.6	Guo et al. (2014)

MP, Mapping Population; DAT, Days After Treatment; PVE, Phenotypic Variance Explained (with respect to mentioned QTL).

## 7 Genome-wide association mapping approach for investigating chromosomal regions related to salinity tolerance

Despite higher efficiency and strong detection power to detect a major variant that influences phenotype, mapping of genes using a bi-parental mapping population faces several challenges, especially where cross incompatibility is a major barrier to developing a successful mapping population. With recent advancements in the Next Generation Sequencing (NGS) and HTP, alternate strategies for mapping genes and QTL have become possible. Genome Wide Association Study (GWAS) is a widely used approach to overcome the shortcomings of bi-parental mapping by utilizing the large genetic variation present in a diverse panel of plants (Tibbs Cortes et al., 2021). GWAS rely on Linkage Disequilibrium (LD), the correlation structure that exists among DNA variants in the candidate genome as a result of historical evolutionary forces, particularly finite population size, mutation, recombination rate, and natural selection (Visscher et al., 2017).

GWAS approaches have been implemented in different plant species to find out the underlying genetics of several abiotic stresses which include drought, salinity, temperature, and Boron toxicity tolerance (Challa and Neelapu, 2018). Major crop-specific achievements in identifying potential QTL related to salt tolerance, important stress indicative parameters, and crop growth stage while screening through GWAS, in recent years are discussed in the following section (**Table 4**).

**Table 4:** Summary of major genome-wide association studies carried out in different crops in recent years.

Crop	Size of diversity panel	Marker system	Significant Marker-trait associations	Crop stage	Major traits	PVE range (%)	Author
Rice	180 accessions	SSR	28	Reproductive	Na^+^, K^+^, Ca^2+^, Mg^2+^ content in stem and leaves, chlorophyll (chl)	5.12-13.37	Warraich et al. (2020)
	155 varieties	SNP	151	Early vegetative	Length, fresh wt., dry wt. of several agronomic traits, and RWC	5.58-21.17	Nayyeripasand et al. (2021)
	553 accessions	SNP	–	Vegetative	Growth parameters through RGB analyses, TR, TUE	–	Al-Tamimi et al. (2016)
	220 accessions	SNP	64	Reproductive	Plant height, ion content, yield parameters	5-15	Kumar et al. (2015)
Wheat	289 elite lines	SNP	118 at low salinity 120 at high salinity	Whole plant growth	Chl and yield related parameters	3.91-17.59	Chaurasia et al. (2020)
	135 accessions	SNP	220	Vegetative	Chl, growth parameters, Na^+^, K^+^ contents	0.10–45.02	Chaurasia et al. (2020)
Maize	305 lines	SNP	53	Seedling	Ca^2+^ in shoot and root	–	Liang et al., 2022)
	445 accessions	SNP	57	Seedling	Plant’s survival rate	5-14	Luo et al. (2019b)
*Brassica napus*	146 accessions	SNP	77	Seedling	Germination vigour, germination rate, relative salt damage index	11.20-17.68	Zhang et al., (2022b)
	228 accessions	SNP	142	Seedling	Germination%, root length, shoot dry weight, seed vigour index	9.6-19.5	Wassan et al. (2021)
	85 inbred lines	SNP	62	Seedling	Shoot fresh weight, shoot dry weight, leaf Na^+^, K^+^, Ca^2+^, leaf Na^+^/K^+^, leaf Na^+^/Ca^2+^	–	Yong et al. (2015)
	505 accessions	SNP	31	Germination and seedling	Germination potential, germination rate, shoot length, root length, dry weight, leaf area, chlorophyll content, relative electrical conductivity	–	Zhang et al. (2022a)
	368 cultivar and inbred lines	SNP	75	Seedling	Root length, shoot length, shoot fresh eight, shoot Na^+^ content	4.21-9.23	Wan et al. (2017)
Soybean	305 accessions	SNP	Three gene-based SNP markers (Salt-20, Salt14056 and Salt11655)	Seedling	Leaf scorch score, chlorophyll content ratio, leaf Na^+^ and Cl^-^ content	–	Do et al. (2018)
	281 accessions	SNP	22 QTL	Germination	Fresh weight, root dry weight	3.83–8.0	Cao et al. (2021)
	121 wild accessions	SNP	21	Germination	germination index, rate, and potential	14.11 - 37.34	Shi et al. (2022)

### 7.1 Rice

Warraich et al. (2020) conducted GWAS in rice at the reproductive stage where 180 diverse accessions were evaluated for 13 morpho-physiological parameters (including ion contents in stem and leaves, grain yield, and salt injury score) and genotyped by genome-wide SSR markers. This study identified 28 significant marker-trait associations, out of which 19 associations were related to ion homeostasis in stems and leaves. GWAS with 155 rice varieties at the early vegetative stage identified 151 significant marker trait associations scattered on 10 chromosomes (Nayyeripasand et al., 2021). The diverse panel was phenotyped with several agronomic parameters such as shoot and root length, the biomass of above and below ground parts, and RWC. A genomic region of 11.26 Mbp on chromosome 1 was found to be colocalised with the QTL region *SalTol1*. A number of candidate genes involved in ion transportation and encoding transcription factors were identified on different chromosomes. Al-Tamimi et al. (2016) used 533 rice accessions (297 *indica* and 257 *aus*) to investigate early response of rice to soil salinity where they phenotyped the accessions for projected shoot area (PSA), absolute and relative growth rate (derived from PSA) using RGB imaging, transpiration rate (TR), and transpiration use efficiency (TUE). Through GWAS, combining these phenotyping data identified new loci affecting TUE on chromosome 11. Another GWAS was successfully implemented in rice to identify SNPs related to 12 different salt tolerance related traits during the reproductive stage under field grown conditions (Kumar et al., 2015). Twenty and 44 SNP loci were identified to be associated with Na^+^: K^+^ ratio and other yield parameters (**Table 4**) observed under salinity, respectively. Widely reported QTL at the seedling stage present on chromosome 1: *Saltol*, was identified to be associated with ion homeostasis at the reproductive stage. Other potential QTL were detected on chromosomes 4, 6, and 7 (Kumar et al., 2015).

### 7.2 Wheat

A recent genome wide association study by Alotaibi et al. (2022) in 289 elite lines of the Wheat Association Mapping Initiative (WAMI) population was conducted under low and high salinity conditions using 15,737 SNP markers at the whole plant growth stage. Seven yield related traits were evaluated and 118 significant marker trait associations at low salinity and 120 at high salinity were found. A multi-locus GWAS in 135 diverse lines at the vegetative stage of wheat revealed novel genomic regions for salinity tolerance (Chaurasia et al., 2020). Out of a total 220 Quantitative Trait Nucleotides (QTNs) identified for 12 salt tolerance related traits, 42 QTNs were found to have significant impacts on 10 salt tolerance traits. Further studies identified 58 candidate genes for the associated genomic regions.

### 7.3 Maize

An association panel comprising 305 maize inbred lines were phenotyped for above and below ground Ca^2+^ concentrations and transport coefficients, and genotyped by SNPs to perform GWAS. Fifty three significant SNPs along with 544 associated genes in the linkage disequilibrium regions were identified under salt treatment. Further expression and genetic variation effects by gene-based association analysis revealed a significant association of a pentatricopeptide repeat protein coding gene *GRMZM2G123314* with Ca^2+^ transport (Liang et al., 2022). Another study by Luo et al. (2019b), investigated the survival rates of 445 maize accessions under salinity stress at the seedling stage. GWAS detected 57 loci significantly associated with salt tolerance, which contained 49 candidate genes.

### 7.4 Brassica

To know the genetic basis of salt tolerance in rapeseed a GWAS was conducted on a diverse panel of 146 accessions using 10,658 high quality SNP markers (Zhang et al., 2022b). In this study 77 SNPs were identified having significant associations with salt tolerance traits, out of which 36 SNPs were associated with three salt tolerance traits (germination vigor, germination rate, relative salt damage index) and gene ontology annotations revealed 19 candidate genes having putative roles in response to salt stress. Using 2,01,817 SNP markers in a GWAS, Wassan et al. (2021) identified 142 SNPs significantly associated with salt tolerance in a diverse panel of 228 lines of *Brassica napus*. They obtained 117 candidate genes associated with 40 SNPs mostly encoding transcription factors, DNA binding proteins, and aquaporins. Further differential expression between salt tolerant and susceptible lines validated ten candidate genes. Sequence analysis of putative candidate genes by Yong et al. (2015) revealed the loss-of-function in coding regions was due to frame shift mutations or formation of premature stop codons. This resulted in differential response in salt tolerant and sensitive lines under stressed condition. Similarly, QTL and candidate genes related to salt stress in both germination and seedling stage in rapeseed were identified by Zhang et al. (2022a). This study reported overexpression of two candidate genes *BnCKX5* and *BnERF3* were associated with increased sensitivity to salt stress. Another GWAS experiment identified 75 SNPs and 38 putative candidate genes associated with salt stress tolerance traits under multiple environments at the seedling stage of brassica (Wan et al., 2017).

### 7.5 Soybean

Do et al. (2018) conducted two GWAS on a diverse panel of soybean accessions to map and validate genomic regions for salt tolerance at the seedling stage. SNPs derived through *SoySNP50K* (Song et al., 2013) from 305 accessions and Whole Genome Resequencing (WGR) of a subset of 234 accessions confirmed a major locus for salt tolerance on chromosome 3. The highest association of three gene-based SNP markers of the known gene, *Glyma03g32900*, on Chr-3: Salt-20, Salt14056, and Salt11655 were found to be consistent in both studies (Do et al., 2018). Another GWAS on 281 diverse soybean accessions at the germination stage by Cao et al. (2021) identified a total of 22 QTL associated with salt tolerance. Four salt tolerance indices related to germination and biomass were used for phenotyping. Two major QTL were identified on chromosomes 5 and 16. Shi et al. (2022) evaluated 121 wild soybean accessions during seed germination under salt stress conditions. A total of 21 SNPs with significant associations with salt tolerance were found to be distributed in Chr- 2, 3, 10, 18, and 19, where Chr- 10 registered the highest number of associations.

## 8 Role of Whole Genome Resequencing for investigating structural variations related to salinity tolerance

Our understanding of the genetic mechanisms underlying salt stress has become even more effective with the recent developments in Whole Genome Resequencing (WGR) and pangenomics. The goal of a WGR experiment is often to identify the differences between a specific individual’s genome and the reference genome. By comparing the sequenced genomes to the reference, a list of mutations unique to each sequenced individual is acquired. These mutations are typically single nucleotide polymorphisms (SNPs) and insertions-deletions (InDels). Substantial rearrangements, including translocations, inversions, and large copy number changes can also be identified using WGR. Findings of noteworthy information related to salt tolerance through WGR studies in various crops are discussed in the following section.

### 8.1 Rice

Jain et al. (2014) performed WGR to analyze two rice cultivars with contrasting responses to salinity stress and identified 401,683 SNPs and 57,656 InDels. They found a total of 614 genes with a higher density of nonsynonymous SNPs and 576 large-effect SNPs in 1247 genes, which might have a role in the contrasting stress response of those rice cultivars. Furthermore, 266 potential genes were detected that could be validated and utilized for the improvement of salt tolerance. Another study with WGR in rice genotypes having differences in salt tolerance identified 2347 nonsynonymous SNPs and 51 frameshift mutations (Subudhi et al., 2020). Additionally, 396 differentially expressed genes with large-effect variants in the coding regions were identified that are associated with various salt tolerance mechanisms.

### 8.2 Chickpea

A WGR study of chickpea genotypes having contrasting tolerance to salinity was carried out by Rajkumar et al. (2021). In total, 920 InDels and 6173 SNPs were found that could distinguish the chickpea genotypes with differing salinity stress responses. Chromosome 4 and chromosome 1 were found to have the highest number and frequency of DNA polymorphisms. However, the least amount of DNA polymorphisms was found on chromosome 5. DNA polymorphisms were discovered in the cis-regulatory motifs of genes related to abiotic stress, which might affect the response to salinity stress by modifying the binding affinity of transcription factors.

### 8.3 Soybean

Further extension of the previously mentioned study by Do et al. (2019) using WGR with a subset of 234 accessions of soybean facilitated the detection of some additional regions on Chr- 1, 8, and 18 which are related to salt tolerance. The region identified on Chr- 8 was predicted as a new minor locus for salt tolerance in soybean. In another study, Patil et al. (2016) conducted a WGR experiment on 106 diverse soybean lines and identified three major structural variants (SV-1, SV-2, and SV-3) and allelic variation in the promoter and genic regions of the *GmCHX1* ion transporter gene associated with salinity tolerance. The presence of Ty1/copia retrotransposon in the given locus was found in SV1, which was manifested by salt-sensitive genotypes as reported by Qi et al. (2014). In contrast, salt-tolerant SV-2 lacked this retrotransposon. Interestingly, the SV-3, which did not contain any retrotransposon, showed a salt-sensitive reaction.

### 8.4 Medicago

The study by Friesen et al. (2014) used WGR to investigate the genetic basis of adaptation under salinity stress in 39 wild accessions of *Medicago truncatula*. They identified candidate genes such as *Medtr3g098090.1*, which is orthologous to *AtCIPK21* in *Arabidopsis thaliana*. *AtCIPK21* is a calcium-dependent protein kinase that plays a role in abscisic acid and jasmonic acid signal transduction pathways. These pathways play critical roles in the regulation of plant responses to various abiotic stresses, including salinity stress. The researchers found that *Medtr3g098090.1* showed differential expression in response to salt stress, with higher expression levels in salt-tolerant accessions. This gene could be a potential target for the genetic improvement of crop plants for increased salt tolerance.

### 8.5 Linseed

To understand the genetic basis of salt tolerance in linseed, WGR has been used to identify QTL for salt tolerance and candidate genes associated with salt tolerance. Zhao et al. (2022) performed WGR on salt-tolerant and salt-sensitive varieties of linseed and a total of 15 candidate genes related to salt tolerance were identified within a 2.597 Mb region on chromosome 1. The study identified two candidate genes for salt tolerance, *Lus.o.m. scaffold91.141* and *Lus.o.m. Scaffold1.14*, which encode WD40 and cytochrome P450, respectively. Previous studies have demonstrated that overexpression of the *Ginkgo biloba WD40* gene improved salt tolerance in poplar (Xin et al., 2021). In addition, Ahmad et al. (2019) found that the candidate gene *Lus o.m. Scaffold1.14* in linseed encodes a cytochrome P50 protein which promotes flavonoid biosynthesis and enhances plant cell resistance by changing osmotic pressure. These findings suggest that these two candidate genes could potentially be targeted for improving salt tolerance in linseed.

## 9 Genomic selection for developing salinity tolerant crops

**Figure 4:**
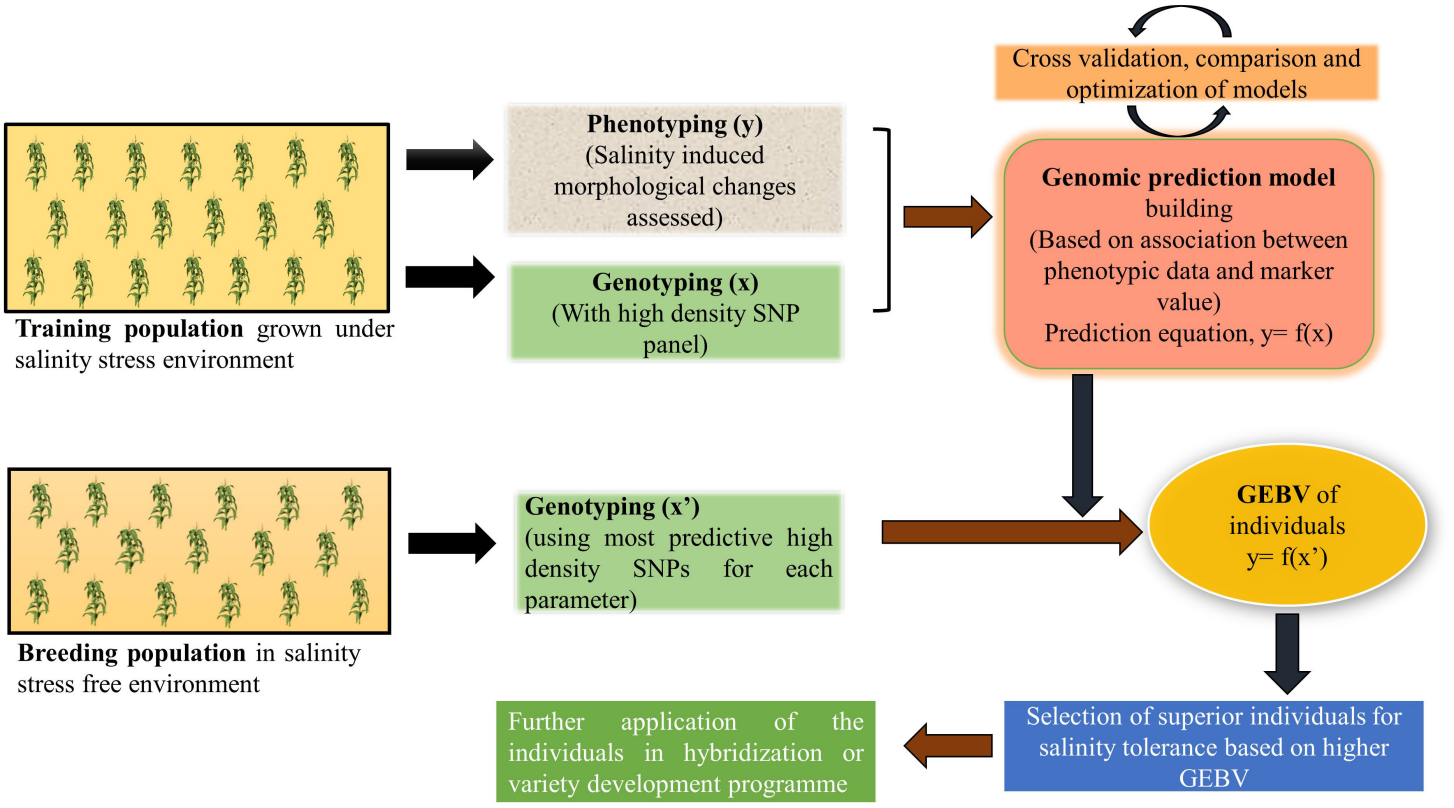
A schematic illustration of genomic selection to improve salinity stress tolerance in crop plants.

In nature, most of the quantitative traits of economic importance like- yield, biotic and abiotic stress tolerance are controlled by a large number of minor effect QTL and MAS fails to capture these small effect alleles (Xu and Crouch, 2008; Bernardo, 2016). To overcome this challenge, a prediction model-based marker strategy called genomic selection (GS) was introduced (Meuwissen et al., 2001). GS uses a training population to develop a prediction model based on its phenotypic and genotypic data and this model in turn used to obtain genomic estimated breeding value (GEBV) of all the individuals of a large breeding population using its genotypic information only (Poland et al., 2012). There are several methods to develop parametric and non-parametric prediction models like- best linear unbiased prediction (BLUP), Bayesian regression, ridge regression, kernel regression, machine learning methods (random forest, support vector machine) (de Los Campos et al., 2013) and their prediction accuracy depends on several factors like- size of the training population (Technow et al., 2013; Endelman et al., 2014), heritability of the trait (Isidro et al., 2015; Duangjit et al., 2016), and marker density for the genotyping population (Zhang et al., 2015). Medina et al. (2020) used eight different GS models to predict the performance of alfalfa under salt stress condition. Prediction accuracy and root mean square due to error (RMSE) values were the criteria to select the best-fitting model. Among rrBLUP, BayesA, BayesB, BayesC, Bayesian Ridge Regression (BRR), Bayesian LASSO (BL), Support Vector Machine (SVM), and Random Forest (RF), SVM outperformed the others with a prediction accuracy of 0.793 for yield. With a negative correlation value r^2^= -0.64 between accuracy and RMSE, SVM proved to be the best for predicting breeding values with high accuracy and low RMSE. Other studies also found machine learning methods to be better over others, as it identifies both additive and non-additive (dominant and epistatic) variance of the trait by capturing SNPs with major effects and complex SNP-SNP interactions (Li et al., 2018; Sandhu et al., 2021a; Enoma et al., 2022). Bartholomé et al. (2022) attempted genomic selection in rice for salt stress related morphological traits and ion mass fractions where they trained the GS model using SNP arrays in a training population of 241 japonica accessions. A high prediction accuracy of 0.25-0.64 for morphological traits and moderate to high accuracy (0.05-0.40) for stress indices were found when this model was used to predict a breeding population. Further cross validation resulted in a strong correlation (r^2 ^= 0.69) in the predictive abilities of a subset of a breeding population with the reference panel under salinity stress conditions. All of these points to the possibility that breeding efforts for developing new lines for salinity-prone environments could become more effective by integrating genomic selection in breeding programs (**Figure 4**).

## 10 Scope of pangenomics

WGR studies based on a single reference genome may not be good enough in identifying Structural Variations (SVs) and do not provide sufficient details for extensive understanding of genetic variants due to the lack of diversity within a species (Bayer et al., 2020; Danilevicz et al., 2020). Considering these limitations, the concept of pangenomics was put forward by the researchers. Pangenomes not only represent more diversity of a species or identify core genes those are present in all individuals, but also identify distinct and variable genes that are missing in the reference genome and in some individuals, respectively (Zhao et al., 2018; Bayer et al., 2020). It also facilitates a multidimensional analysis of polymorphism and SVs within and across genomes to simultaneously investigate the genomic variations among individuals of a species or higher taxonomic groups.

Pangenomes have been compiled for many major crop species such as maize, rice, hexaploid wheat, soybean, and *Brassica oleracea*, but have yet to be fully explored in terms of connecting genetic and phenotypic variations, particularly about salt tolerance (Hirsch et al., 2014; Li et al., 2014; Golicz et al., 2016; Montenegro et al., 2017; Zhao et al., 2018). To combat harsh environmental conditions, researchers are still working to mine certain superior genes related to salinity tolerance from wild relatives through forward genetics or reverse genetics approaches. The identification and mapping of salinity-tolerant genes in important crop species have not yet been reported from a pangenome analysis (Ullah et al., 2022). This pangenome idea along with high-throughput third-generation sequencing offers a reliable platform to recapture deleted genes, identify new genes, and increase our understanding of the dynamics and architecture of the genome. Therefore, considering the power of pangenome analysis to address salinity stress, research efforts are required to mine novel genes in exotic germplasms.

## 11 Conclusions and future prospects

Irrigation with recycled water, improper fertilization, and deterioration of agricultural lands are gradually increasing the threat of soil salinization (Jha et al., 2019). Consequently, salinity being one of the major abiotic stresses, causes significant yield loss in agricultural crops worldwide. To mitigate crop yield loss caused by the rising challenge of salinity stress, various strategies have been embraced. Interdisciplinary research advances in various model plants as well as crop plants have improved our understanding of the complex molecular mechanisms and underlying genetics of salinity tolerance. Similarly, advances in genomic resources have allowed for the identification of salinity tolerance QTLs and genes through bi-parental and genome wide association mapping studies. Furthermore, availability of crop genome sequences, genome re-sequencing approaches, and pangenome assemblies can greatly facilitate in discovery of genomic regions, structural genomic variants or haplotypes contributing to salinity tolerance across the whole genome.

Increasing facilities capable of HTP has improved the screening of germplasms with enhanced phenotypic accuracy and efficiency which has been proven highly effective for selecting potential salinity tolerant crop plants (Al-Tamimi et al., 2016). Emerging genome editing techniques, such as CRISPR/Cas9, are powerful tools for the validation of candidate genes and knocking out negative alleles associated with salt susceptibility (Kumar and Sharma, 2020; Luo et al., 2021). Future research studies may explore special traits and structures such as salt glands, papillae etc. present across various plant gene pools and harness their potential towards improving salinity tolerance (Marcum et al., 1998; Yamamoto et al., 2016; Jha et al., 2019; Spiekerman and Devos, 2020; Mondal et al., 2023). Deciphering different pathways involved in salt tolerance, understanding genetics through molecular mapping, embracing conventional breeding approaches along with emerging breeding tools can help improve genetic gain under increasing salinity stress, leading to more resilient crops for the future.

## Author contributions

All authors contributed through writing and editing the manuscript and they approved the submitted version.
